# Health Promoting Lifestyle Behaviors in Menopausal Women: A Cross-Sectional Study

**DOI:** 10.5539/gjhs.v8n8p128

**Published:** 2015-12-17

**Authors:** Fereshte Shabani Asrami, Zeinab Hamzehgardeshi, Zohreh Shahhosseini

**Affiliations:** 1 Student Research Committee, Mazandaran University of Medical Sciences, Sari, Iran; 2 Department of reproductive health and Midwifery, Nasibeh Nursing and Midwifery faculty, Mazandaran University of Medical Sciences, Sari, Iran; 3 Traditional and Complementary Medicine Research Center, Mazandaran University of Medical Sciences, Sari, Iran

**Keywords:** health promoting behaviors, menopausal women, health promotion

## Abstract

**Background:**

Determining health promoting lifestyle behaviors of age-specific groups of women provides valuable information for designing health promotion intervention programs. Hence the present study was conducted to assess health promoting lifestyle behaviors in menopausal women.

**Methods:**

The present descriptive cross-sectional study examined health promoting lifestyle behaviors in 400 menopausal women admitted to health care centers in Neka city-north of Iran-from March 2015 to July 2015. Health promoting lifestyle behaviors were evaluated using a demographic characteristics form and the Health Promoting Lifestyle Profile II (HPLP II) through simple convenience sampling. Data were analyzed in SPSS version 18 using descriptive and inferential statistics at the significance level of P<0.05.

**Results:**

The mean score of participants’ health promoting lifestyle behaviors was 136.43±19.61, ranging from 88 to 194. The logistic regression test revealed women’s health promoting lifestyle behaviors to be significantly related to their place of residence (P=0.009, odds ratio=1.73) and their spouse’s level of education (P=0.027, odds ratio=0.58). The Pearson correlation test showed significant relationships between mean score of the six sub-scale of health promoting lifestyle behaviors with each other (P<0.001).

**Conclusion:**

These findings have implications for addressing the role of men to promote health promoting lifestyle behaviors among rural menopausal women.

## 1. Introduction

Menopause is a developmental stage experienced by all women as they age. According to statistics, the world’s population of menopausal women was 467 million in 1990. This population is expected to reach 1000.2 million women by 2030 (McKinlay, Brambilla, & Posner, 1992; Nichols et al., 2006; Randhawa & Sidhu, 2014). In Iran, there will be about five million menopausal women by 2021 and their life expectancy is currently about 70 years. Thus, it is expected they will to live nearly one third of their lives after menopause (Ayatollahi, Ghaem, & Ayatollahi, 2005; Fallahian, 2004; Statistical Centre of Iran, 2011).

Menopausal changes that occur in women effect on different aspects of their health status like; reduce their physical activity, lead to sleep disorders, reduce their social activities and negatively related to their health promoting behaviors (Poomalar & Arounassalame, 2013). Therefore, finding an appropriate and harmless health promoting solution to overcome these problems is essential (Gabriel, Mason, & Sternfeld, 2015; Tehrani, Ghobadzadeh, & Arastou, 2007).

Many of the serious complications that women face during menopause are caused by their own poor lifestyles and their lack of knowledge about self-care (Heidenreich, 2014; Kim, Lee, & Johnson, 2015). To minimize these complications, efforts should be made to promote menopausal women’s health. It is obvious that the promotion of health requires adherence to specific associated health behaviors (Baheiraei et al., 2011; Yeom, 2014).

Health promoting lifestyle behaviors are as active approaches to promote personal welfare and perceptions to maintain or enhance the level of wellness, self-actualization and fulfillment of the individual (Shaheen, Nassar, Amre, & Hamdan-Mansour, 2015; Sousa, Gaspar, Vaz, Gonzaga, & Dixe, 2015). They include behaviors through which the individual attempts to follow a proper diet, attempts to engages in regular exercise, attempts to avoids high-risk behaviors and drug use, attempts to protects herself against accidents, and pays attention to diverse physical dimensions, controlling emotions, feelings, and thoughts, defending against mental tensions and problems (Pullen, Walker, & Fiandt, 2001).

Some studies conducted by means of Health Promoting Lifestyle Profile II (HPLP II) around the world show that health promoting lifestyle behaviors vary in different societies based on their particular socio-cultural-economic contexts. One study reported that the highest score of health promoting lifestyle behaviors in rural women was detected in nutritional status and the lowest in physical activity (Pullen et al., 2001). Meanwhile, another study conducted on Hispanic women found the highest score have been obtained in spiritual growth and the lowest in physical activity (Hulme et al., 2003). Also, in Korean farmers the highest score of health promoting lifestyle behaviors was shown to have related to nutrition, followed by spiritual growth, and the lowest to have been obtained in physical activity (Park, Kwon, & Oh, 2009). Finally, another study on nurses showed the highest score of health promoting lifestyle behaviors was detected in nutrition dimension and interpersonal relationships and the lowest were related to physical activity and stress management (Edrisi et al., 2013).

Given the importance of health promoting lifestyle behaviors in menopausal women, the present study was conducted to assess these behaviors in menopausal women admitted to health care centers with the aim of addressing health-oriented programs for menopausal women.

## 2. Method

The present descriptive cross-sectional study examined health promoting lifestyle behaviors in 400 menopausal women admitted to health care centers in Neka city-north of Iran-from March 2015 to July 2015. The women with at least one year miss period of menstruation were selected through simple convenience sampling. Data were collected using a demographic characteristics form and the Health Promoting Lifestyle Profile II (HPLP II) measuring 52 health promoting lifestyle behaviors in a four point Likert scale type for each item ranging from 1(never) to 4(routinely). The HPLP II includes six sub-scales: health responsibility (9 items), physical activity (8 items), nutrition (9 items), spiritual growth (9 items), interpersonal relationships (9 items), and stress management (8 items). The overall score of the instrument range from 52 to 208, where each domain has its own separate score and higher scores indicate a better adherence to health promoting behavior. In this way, scores 52-103 means a low level of adherence to these behaviors and scores 104-155 and score above 156 imply a moderate and high level, respectively.

This instrument was designed by Walker, Sechrist and Pender in 1987 to measure health promoting behaviors (Walker, Sechrist, & Pender, 1987). It has been translated into many languages and has an acceptable validity and reliability (Pérez-Fortis, Díez, Sara, & Padilla, 2012; Pinar, Celik, & Bahcecik, 2009; Sousa, Gaspar, Fonseca, Hendricks, & Murdaugh, 2015; Wang et al., 2007). A number of Iranian studies have evaluated psychometric properties of the Persian version of this profile too (Baheiraei et al., 2011; Mohamadian, Ghannaee, Kortdzanganeh, & Meihan, 2013).

After submitting signed informed consent form, the participants were invited to complete the HPLP II. The ethical consideration of the research was observed from the Ethics Committee of Mazandaran University of Medical Sciences (No.93-179), obtaining letters of introduction from the relevant organizations and maintaining the confidentiality of the data.

Data were analyzed in SPSS version 18 using descriptive and inferential statistics including the independent t-test, the analysis of variance, Pearson correlation test, and the logistic regression analysis at a significance level of P<0.05.

## 3. Results

Participants’ mean age and their mean age at menopause were reported as 57.53±7.63 and 47.78±5.12, respectively. A total of 44% of participants lived in cities, and the rest lived in rural areas. Table 1 presents the demographic characteristics of participants.

**Table 1:** Socio-demographic characteristics of the participants

Age(yr)*	57.53±7.63	

Menopausal age(yr)*	47.78±5.10	
Residency of location**	Urban	176(44)
	Rural	224(56)
Marital status**	Married	339(84.8)
	Widow/Divorced	61(15.2)
Level of education**	Primary	354(88.5)
	Secondary	39(9.8)
	Academic	7(1.7)
Employment Status**	Household	385(96.3)
	Employed	15(3.7)
Family structure**	Living with husband	326(81.5)
	Living with children	48(12.1)
	Living alone	26(6.4)
Education of husband**	Primary	286(71.5)
	Secondary	96(24)
	Academic	18(4.5)
Economic status**	Independent	319(79.8)
	Dependent	81(20.2)

* Mean±SD;

** Frequency (Percent).

According to the results, mean score of health promoting lifestyle behaviors was 136.43±19.61, ranging from 88 to 194. The highest mean score in the subscales was 25.88±4.86 for nutrition sub-scale followed by interpersonal relationship (25.82±5.02) and spiritual growth (25.45±4.46), and the lowest was 16.12±4.30 for physical activity. Table 2 presents the mean score of health promoting lifestyle behaviors by place of residence.

**Table 2:** Mean and standard deviation of health promoting behaviors score by location of residency

	Urban N=176	Rural N=224	t	Total N=400
	
	Mean±SD	Mean±SD		Mean±SD
Health Responsibility	23.63±4.86**	23.53±4.10**	0.88	23.57±4.45**
Physical Activity	15.76±4.37***	16.41±4.24***	-1.5	16.12±4.30***
Nutrition	27.64±5.28**	24.50±3.99**	6.55*	25.88±4.86**
Spiritual Growth	25.69±4.79**	25.27±4.17**	0.91	25.45±4.46**
Interpersonal Relationship	26.69±5.67**	25.13±4.34**	3.00*	25.82±5.02**
Stress Management	19.78±4.30***	19.43±3.29***	0.88	19.59±3.77***
Total Score	139.17±21.75	134.27±17.5	2.43*	136.43±19.61

* *P*<0.05 was considered statistically significant;

** Total score ranged 9-36;

*** Total score ranged 8-32.

The results also showed that health promoting lifestyle behaviors are significantly related to women’s level of education (F=4.81, P=0.003), their spouse’s level of education (F=5.62, P<0.001), and their location of residency (t=2.43, P=0.011). However, the score of health promoting lifestyle behaviors was not found to be significantly related to the women’s age (r=0.22, P=0.655), age at menopause (r=0.28, P=0.575), marital status (t=1.67, P=0.097), employment status (t=0.57, P=0.568), family structure (F=1.71, P=0.163), and economic status (t=0.57, P=0.567). The Pearson correlation test showed significant relationships between mean score of the six sub-scale of health promoting lifestyle behaviors with each other (P<0.001).

To provide a better interpretation of the relationships discovered, the Binary logistic regression analysis (cut off point ≥ 130 score was considered as positive history of health promoting lifestyle behaviors) was conducted. The results showed that the score of health promoting lifestyle behaviors in menopausal women related to their location of residency (P=0.009, odds ratio=1.73) and their spouse’s level of education (P=0.027, odds ratio=0.58). It means the mean score of health promoting lifestyle behaviors was significantly higher in urban women compared to rural ones and women who also had better educated spouses had a better adherence to health promoting lifestyle behaviors (Table 3).

**Table 3:** Logistic regression analysis for variables predicting health promoting behaviors in menopausal women

	B	Std.Error	Sig.	EXP(B)	95% Confidence Interval for B	

					Lower	Upper
Location of residency	0.552	0.210	0.009	1.736	1.151	2.619
Level of education	-0.639	0.365	0.080	0.528	0.258	1.078
Husbands’ level of education	-0.534	0.242	0.027	0.586	0.365	0.942
**Constant**	0.970	0.349	0.005	2.637		

## 4. Discussion

The mean score of health promoting lifestyle behaviors of our participants was reported in moderate range, which is consistent with other studies conducted in Iranian perimenopausal middle-aged women (Enjezab, Farajzadegan, Taleghani, Aflatoonian, & Morowatisharifabad, 2012; Sehhatie, Mirghafourvand, & Momeni, 2015) and younger Iranian nurses (Edrisi et al., 2013). It seems women necessitate a greater attention of policy makers and health care services to promote health promoting training programs targeting them. Although In a study among Taiwanese women the mean of the overall score for health promoting lifestyle behaviors fell within the low range (Lee & Wang, 2005), possibly indicating cultural differences in health behaviors between Iranian and Taiwanese women, thus justifying the need for conducting further studies in different countries.

In attention to numerous studies have shown physical activity has positive effects on menopausal symptoms (Daley et al., 2015; Kim, Cho, Ahn, Yim, & Park, 2014; Sternfeld et al., 2014) and low physical activity score in this research along with other studies (Hulme et al., 2003; Kirag & Ocaktan, 2013; Park et al., 2009; Pullen et al., 2001), the need for interventional programs for menopausal women is recommended.

In the present study, in line with some studies, the results showed significantly higher scores of health promoting lifestyle behaviors in urban women compared to rural ones (Park, 2002), although in some studies there is no evidences to support this (Lee, 2009; Query, 2014). This differences in results may be due to the fact that rural women, in north of Iran, traditionally, are more involved in agricultural work (Sehhatie et al., 2015) and may less pay to attention to health promoting behaviors, so more research is necessary before a conclusion can be made.

Our finding regarding to the relationship between women’s health promoting lifestyle behaviors and their spouse’s level of education may be considered as a noticeable issue in this project. As, especially, in developing countries, men could be decision-makers in issues related to women’s health (Khani, Banaem, Mohammadi, Vedadhir, & Hajizadeh, 2014; Mboane & Bhatta, 2015; Speizer, Whittle, & Carter, 2005), their role must be kept in mind in policies related to health promotion of their wives and the less educated men appear to require more careful training and planning in this regard.

Despite some studies have shown more educated women have higher score in health promoting lifestyle behaviors (Arras, Ogletree, & Welshimer, 2006; Bond, Jones, Cason, Campbell, & Hall, 2002; Meihan & Chung-Ngok, 2011; Sohng, Sohng, & Yeom, 2002), in our study there is no evidences to support this (El Mokadem, 2013). This may be because the majority (88.5%) of participants in this study, in accordance with normal population of menopausal women in Iranian society, (Statistical Centre of Iran, 2011) had primary education. Also it is not clear that someone with higher school education have an understanding of the importance of certain health promoting behaviors? More population-based studies with considering determinants of health promoting behaviors may be helpful in the future.

In conclusion, it seems the level of education of women’s husbands can predict menopausal women adherence to health promoting behaviors. Hence, the less educated men appear to require more careful training and planning in this regard. Moreover, the poorer adherence to health promoting lifestyle behaviors in rural women necessitate a greater attention of health policy makers to holding health training programs targeting this group of menopausal women.

One limitation of the present study is that it was conducted on women admitted to health care centers, and its results are, therefore, only applicable to this group of women. Future studies are recommended to be conducted through population-based design research in order to visit menopausal women in their place of residence.
